# Familial spinocerebellar ataxia type 3: A case report of multi-generational presentation

**DOI:** 10.1097/MD.0000000000043812

**Published:** 2025-08-08

**Authors:** Ji Wang, Zheng Liu, Chao Shi, Chunhua Pan, Junting Chen, Ziyi Zhao, Dan Yang, Hao Huang

**Affiliations:** a Department of Neurology, Affiliated Hospital of Zunyi Medical University, Zunyi, China.

**Keywords:** family, gene mutation, MJD, SCA3, spinocerebellar ataxia type 3

## Abstract

**Rationale:**

Spinocerebellar ataxia type 3 (SCA3), also known as Machado–Joseph disease, a rare autosomal dominant neurodegenerative disorder caused by cytosine–adenine–guanine repeat expansions in ATXN3, lacks effective therapies. This case report highlights the clinical and genetic features of a family with 5 affected members to emphasize the challenges in diagnosis, management, and the need for targeted therapies.

**Patient concerns:**

A 33-year-old male proband presented with progressive gait instability, limb incoordination, and dysarthria for more than 2 years. The symptoms worsened in cold weather and were accompanied by head swelling and muscle weakness. The patient reported a strong family history of similar neurological symptoms across the 3 generations.

**Diagnosis:**

Clinical evaluation revealed cerebellar ataxia, a wide-based gait, and impaired coordination. Brain MRI revealed bilateral cerebellar atrophy. Genetic testing confirmed the presence of a pathogenic ATXN3 allele with 78 cytosine–adenine–guanine repeats (normal range: ≤49), which is consistent with SCA3. Familial genetic analysis identified identical mutations in 4 additional relatives.

**Interventions:**

Supportive treatment included improvement in circulation, neuroprotective agents, and symptomatic management. No disease-modifying therapies were administered, owing to their limited availability.

**Outcomes:**

The patient’s condition did not improve during hospitalization, reflecting the progressive nature of the SCA3. Similar outcomes were observed in affected family members.

**Lessons:**

Early genetic testing is critical for a definitive diagnosis, especially in familial cases. The lack of effective therapies underscores the urgency of clinical trials that target polyglutamine toxicity. Multidisciplinary care and patient education are essential for the management of this debilitating disease.

## 1. Introduction

Spinocerebellar ataxia type 3 (SCA3), also known as Machado–Joseph disease (MJD), is a progressive neurodegenerative disease caused by cytosine–adenine–guanine (CAG) repeat expansion of the ATXN3 gene encoding ataxia protein-3. It is the most common dominantly inherited ataxia worldwide, affecting approximately 1 in 50,000 to 100,000 individuals, and is also 1 of 9 polyglutamine (polyQ) neurodegenerative diseases, with a prevalence second only to Huntington disease in polyQ diseases. This group of diseases originates from abnormal amplification of triplet CAG in the coding regions of different and unrelated genes. This amplification is converted into a glutamine extension in the coding protein ataxin-3 in MJD. The CAG repeat number of these genes in the normal population is less than a certain threshold (14–44 repeats), whereas the CAG repeat number in SCA3 patients is a complete mutation (52–86 repeats).^[1–4]^ SCA3 is characterized by cerebellar ataxia, which is the main symptom that progressively worsens. It is characterized by unstable gait, poor body balance, and poor motor coordination during walking. It may be accompanied by extraocular muscle paralysis, dysphagia, pyramidal tract signs, extrapyramidal system signs, tongue muscle tremors, muscle atrophy, and muscle tone disorders.^[5–7]^ However, this type of disease lacks effective treatment methods and cannot prevent disease progression; therefore, only routine symptomatic treatment can be used, and the patient’s condition gradually progresses, often causing great physical, mental, and economic burdens. We report 5 cases of SCA3 in a family treated at our hospital.

## 2. Case report

**Figure 1.**
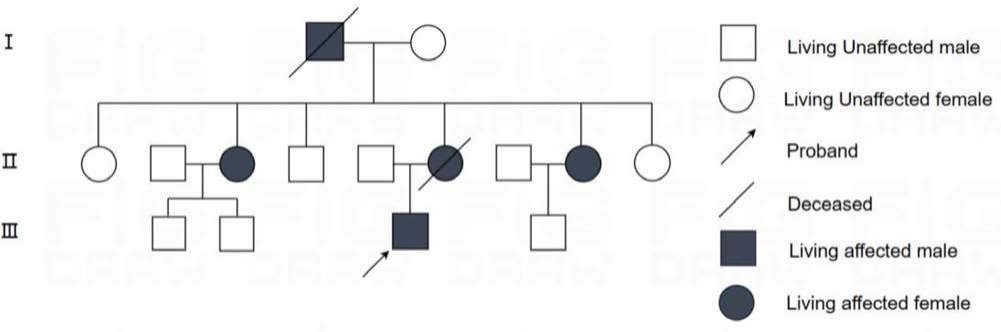
Presents a pedigree chart of a family affected by SCA3, using standard symbols: circles for females, squares for males, filled shapes for individuals with SCA3, and open shapes for unaffected members. The proband (the first diagnosed case in the family) is typically marked with an arrow or highlighted symbol. Diagonal lines denote deceased individuals. This chart visually summarizes the distribution of the condition within the family lineage. SCA3 = spinocerebellar ataxia type 3.

**Figure 2.**
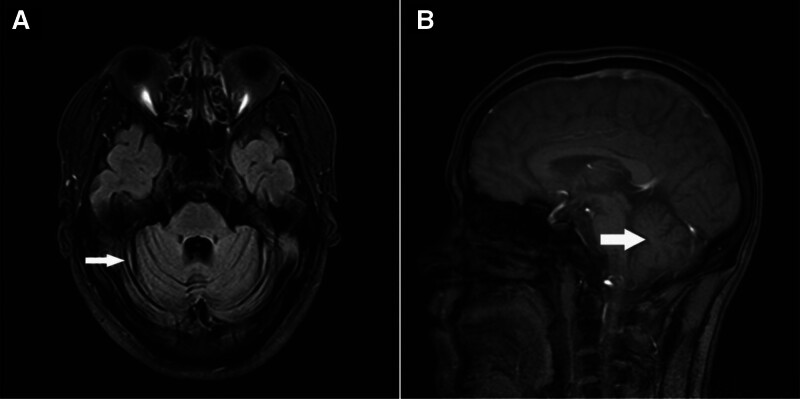
(A and B) Head MRI results of the proband: the brain parenchyma shows no abnormal signals, with a clear demarcation between the gray and white matter. The size and signal of the ventricular system were normal. There was bilateral widening of the cerebellar sulci, whereas the cerebral sulci and cisterns appeared normal. The midline structures were not displaced, and the brainstem contour was well defined. MRI = magnetic resonance imaging.

The proband, male, 33 years old, had an insidious onset and was admitted to the hospital with the chief complaint of “walking incoordination of both lower limbs for 2 years and aggravation for 3 months.” Two years prior to admission, the patient gradually developed unsteady walking in both lower limbs without an obvious trigger, falling left and right, and a feeling of jumping in both lower limbs while going downhill. The above symptoms were significantly aggravated in cold weather, they were not paid attention to, and they did not visit the hospital for treatment. In the past 2 years, the above symptoms were slowly aggravated; and 3 months ago, the right lower limb turned outward while walking, occasionally vomiting words, accompanied by head swelling, and acid and soft legs of both lower limbs. No headache, fever, cough when drinking water, unconsciousness disorder, limb numbness, urine or bowel disorder. For further diagnosis and treatment, the patient was referred to the outpatient department of our hospital, which was admitted to our department for “ataxia reason.” His spirit, diet, and sleep were normal, urine was normal, and there was no significant increase in weight. The past and personal history were not special; there were 5 people in 3 generations in the family with a similar medical history, and the incidence rate was advanced by generation (Fig. 1). Physical examination revealed a blood pressure of 138/95 mm Hg. No significant positive signs were observed on the cardiopulmonary abdomen. Physical examination of the nervous system: clear mind, relevant to the question, round and large pupil, diameter of approximately 3 mm, limited abduction of both eyes, white approximately 3 to 4 mm, and no nystagmus. Neck soft, no resistance; The muscle strength and muscle tension of the limbs were normal, the movements of the fingers and nose and rotation were poor, and the tests of the heel, knee and shin on both sides were negative. It is difficult to walk in a straight line and the step base is wide. Closed eyes have difficulty standing up with positive signs (open eyes, closed eyes positive), the body falls to the right, physiological reflexes are present, tendon reflexes in both lower limbs are symmetrically brisk, and bilateral pathological signs are negative. Meningeal stimulation was negative. Auxiliary examination Routine blood and clinical biochemical examinations were normal. Electrocardiogram revealed no abnormalities. Chest CT revealed fibrosis and calcification in the upper lobe of the right lung. Brain MRI revealed bilateral cerebellar atrophy and no abnormalities in the brain parenchyma (Fig. 2A and B). Cervical MRI revealed cervical degenerative changes, spinal stenosis, and mild degeneration of the thoracic vertebrae with multiple pyramidal hummer nodules. The gene detection results showed that the number of (CAG)n repeats in SCA3-related genes was 33, which belonged to the normal range, and 78, which belonged to the total mutation range (Table 1). Matching the SCA3 mutation profile. A combined medical history, head MRI examination, and delivery genetic testing were considered spinocerebellar ataxia type 3 (SCA3). After hospitalization, circulation, nutritional nerves, and corresponding symptomatic support treatment actively improved, but the patient’s condition did not improve during hospitalization.

**Table 1:** The genetic test report indicates that SCA3 gene locus 2 exhibits 78 CAG repeats (normal range ≤ 44), confirming a full mutation positive result and suggesting genetic risk for spinocerebellar ataxia type 3 (SCA3).

Gene (reference sequence)	Region	The locus 1	The locus 2	Conclusion
SCA1:ATXN1	(CAG)n	26 (normal)	29 (normal)	Negative
SCA2:ATXN2	(CAG)n	22 (normal)	22 (normal)	Negative
SCA3:ATXN3	(CAG)n	33 (normal)	78 (full mutation)	Positive

SCA3 is an autosomal dominant inherited disorder, where a single pathogenic mutation is sufficient to cause the disease. No pathogenic mutations were detected in the other tested genes (SCA1/2).

CAG = cytosine–adenine–guanine, SCA1 = spinocerebellar ataxia type 1, SCA2 = spinocerebellar ataxia type 2, SCA3 = spinocerebellar ataxia type 3.

## 3. Discussion

SCA3 mainly affects the cerebellum and its afferent and efferent connecting fibers, and is the most common genetic ataxia in the world, affecting approximately 1:50,000 to 100,000 people. It is also 1 of the 9 polyglutamine (polyQ) neurodegenerative diseases, with a prevalence second only to Huntington disease in polyQ disease. This group of diseases originates from the abnormal amplification of triplet CAG in the coding regions of different and unrelated genes. This amplification is converted into glutamine extension in the coding protein ataxin-3 in MJD. The CAG repeat number of these genes in the normal population is less than a certain threshold, whereas the CAG repeat number in SCA3 patients is a complete mutation.^[1–4]^ SCA3 often occurs in adulthood, with an average onset age of 30 to 50 years old and a slow progression. The clinical symptoms are diverse and include cerebellar ataxia, Parkinson syndrome, muscle tone disorders, and lower motor neuron disease. Some patients also exhibit non-motor symptoms, such as sleep, emotional disorders, pain, spasms, autonomic nervous system disorders, cognitive impairment, psychiatric symptoms, and olfactory impairment. The clinical symptoms of various subtypes may be similar or overlapping, and the same family and type may have different manifestations, which can easily cause difficulties in diagnosing SCA3.^[5–8]^ Genetic testing helps with early diagnosis and determination of specific types of SCA, providing evidence and data for symptom diagnosis, prenatal diagnosis, and gene therapy.^[9]^ Therefore, genetic testing has become the primary method for distinguishing between different subtypes. The patient in this case had typical cerebellar symptoms, similar to most typical patients, with an early onset age and genetic predisposition. Some SCA3 patients may exhibit signs of cerebellar or brainstem atrophy on cranial MRI, and may also exhibit completely normal cranial imaging.

Some studies suggest that this group of diseases originates from the abnormal amplification of triplet CAG in the coding regions of different and unrelated genes. This amplification is converted into a glutamine extension in the coding protein ataxin-3 in MJD. The CAG repeat number of these genes in the normal population is less than a certain threshold, whereas the CAG repeat number in SCA3 patients is a complete mutation. The number of CAG replicates in the Chinese population was 13 to 49, with amplification abnormalities occurring when the number of replicates exceeded 50. The earlier the age of onset, the more amplification times and the more severe the condition. However, the specific mechanism of this disease is still unclear so far.^[8,10–14]^ The proband of this family was a 33-year-old male with slow onset and gradual progression, mainly characterized by cerebellar ataxia. Brain magnetic resonance imaging (MRI) revealed cerebellar atrophy with a clear family history of hereditary diseases. The other patients in this family had similar clinical manifestations and disease progression, and the age of onset gradually increased. Both males and females were diagnosed with this disease. Genetic testing indicated that the number of CAG amplifications was >60, which is consistent with the diagnosis of SCA3.

Some researchers even believe that neuronal damage may precede the onset of clinical symptoms.^[8]^ Previous studies have shown that the serum NSE concentration of SCA3 patients is significantly higher than that of the normal control group and is positively correlated with the disease course and ICARS score, indicating that the longer the disease course of SCA3, the higher the serum NSE level of patients with more severe conditions. This mechanism may be due to the increased number of CAG repeats in the exon region of SCA3, which encodes continuous glutamate residues. This toxicity leads to neuronal degeneration and damage, resulting in the outflow of intracellular substances. These pathological changes persisted throughout the course of the SCA3. Some researchers believe that neuronal damage may occur earlier than the clinical symptoms. Studies have also found that synaptic loss in vivo is associated with the severity of SCA3, indicating that SV2APET may be a clinical biomarker for the progression of SCA3 disease.^[14]^

Currently, there are no effective treatments for spinocerebellar ataxia.^[15]^ In recent years, efforts have been made to understand the molecular pathogenesis of this disease, identify prognostic disease biomarkers, and develop treatment strategies. Studies have shown that T1 to 11 and JMF1907 alleviate pontine neuronal death, cerebellar transcriptional inhibition, and ataxia symptoms in SCA3 transgenic mice induced by mutated ataxia protein-3-Q79 by enhancing proteasome activity and reducing the protein levels of polyglutamine-amplified ataxia-3-Q79 in the pons and cerebellum. Therefore, T1 to 11 and JMF1907 could be used as therapeutic agents for SCA3.^[16]^ Multidisciplinary care and patient education are essential for the management of this debilitating disease. Multidisciplinary care should include: physical therapy 3x/wk for gait training,^[17]^ speech therapy for dysarthria,^[1]^ and genetic counseling for at-risk relatives.^[4]^ Other studies have reported severe but modifiable defects in oligodendrocyte maturation caused by the toxic function acquisition of mutant ATXN3 in the early progression of SCA3 disease, which are rescued by anti ATXN3 antisense oligonucleotides (ASO) in transcription, biochemistry, and function.^[15]^ This indicates the need to consider non-neuronal targets for the treatment of neurodegenerative diseases in the future. Unfortunately, most of these strategies have only been evaluated in preclinical settings and have not been tested in clinical trials. However, many MDJ symptoms respond to the treatment. For example, studies have shown that 1 HzrTMS and iTBS interventions targeting the cerebellum can effectively improve ataxia symptoms in SCA3 patients.^[17]^ Some patients have signs of Parkinson disease, and the use of dopamine drugs may improve this condition. Many MJD patients with restless legs syndrome respond to dopaminergic drugs. Abnormal sleep patterns and daytime drowsiness are common complaints of MJD, which can benefit from parasympathetic drugs, such as amphetamines and modafinil. Some patients may find that amantadine is beneficial for their alertness levels; however, the pharmacological basis of this reaction remains unclear. Therefore, it is necessary to continuously strive to develop high-quality and robust preclinical studies, translate the results obtained into clinical trials, and provide hope to the patients and their families.

## Acknowledgments

The authors thank the Medical Research Union Fund for High-quality health development of Guizhou Province (2024GZYXKYJJXM0102).

## Author contributions

**Data curation:** Chunhua Pan, Junting Chen, Ziyi Zhao.

**Formal analysis:** Chunhua Pan, Junting Chen, Dan Yang.

**Supervision:** Hao Huang.

**Validation:** Hao Huang.

**Writing – original draft:** Ji Wang, Zheng Liu.

**Writing – review & editing:** Ji Wang, Chao Shi.
